# 

**Published:** 1899-03

**Authors:** 


### CONTENTS.

ORIGINAL ARTICLES: PAGE

"I Approve of the Organization of a Veterinary Contingent to the Army. "(Signed) RUSSELL A. ALGER, Secretary of War" . . . . . . 133
Examinations for Soundness. By J. E. RYDER, D.V.S. . . . . . . . 140
A Campaign of Education. By J. M. EMMERT, M.D. . . . . . . . 145
Pseudo-scab. By S. D. BRIMHALL, V.M.D. . . . . . . . . . 150
Practicability of the Serum-Therapy in the Treatment of Hog-cholera. By W. B. NILES, D.V.M. . . . . . . . . . . . . . 151
Veterinarians as Sanitarians. By G. B. BLACKMAN, D.V.S. . . . . . . 155

ABSTRACTS FROM FOREIGN JOURNALS . . . 160

REPORTS OF CASES . . . . . 163

THERAPEUTIC NOTES . . . . . 167

EDITORIAL . . . . . . . 170

SOCIETY PROCEEDINGS . . . . . 173

PERSONALS . . . . . . . 197

LIBRARY

SURGEON GENERAL'S OFFICE

